# Hepatitis B and hepatitis D virus infections in the Central African Republic, twenty-five years after a fulminant hepatitis outbreak, indicate continuing spread in asymptomatic young adults

**DOI:** 10.1371/journal.pntd.0006377

**Published:** 2018-04-26

**Authors:** Narcisse Patrice Komas, Sumantra Ghosh, Mariama Abdou-Chekaraou, Pierre Pradat, Nasser Al Hawajri, Alexandre Manirakiza, Gina Laure Laghoe, Claudine Bekondi, Ségolène Brichler, Jean-Omer Ouavéné, Abdoulaye Sépou, Brice Martial Yambiyo, Jean Chrysostome Gody, Valentin Fikouma, Athénais Gerber, Natali Abeywickrama Samarakoon, Dulce Alfaiate, Caroline Scholtès, Nora Martel, Frédéric Le Gal, Hugo Lo Pinto, Ikram Amri, Olivier Hantz, David Durantel, Jean-Louis Lesbordes, Emmanuel Gordien, Philippe Merle, Tudor Drugan, Christian Trépo, Fabien Zoulim, Jean-Claude Cortay, Alan Campbell Kay, Paul Dény

**Affiliations:** 1 Laboratoire des hépatites virales, Institut Pasteur de Bangui, Bangui, Central African Republic; 2 INSERM, U1052, UMR CNRS 5286, Centre de Recherche en Cancérologie de Lyon, Lyon, France; 3 Service de Microbiologie Clinique, Hôpital Avicenne, Groupe des Hôpitaux Universitaires Paris Seine Saint-Denis, Assistance Publique–Hôpitaux de Paris, Bobigny, France, Université Paris 13/Sorbonne Paris Cité, UFR Santé Médecine Biologie Humaine, Bobigny, France; 4 Center for Clinical Research, Croix-Rousse Hospital, Lyon, France; 5 Hospices Civils de Lyon, Lyon, France/Université de Lyon I, Lyon, France; 6 Agence de Biomédecine, Saint Denis, France; 7 Service d’Épidémiologie, Institut Pasteur de Bangui, Bangui, Central African Republic; 8 Service de Médecine Interne, Hôpital de l’Amitié, Avenue Indépendance, Bangui, Central African Republic; 9 Service de Gynécologie Obstétrique, Hôpital Communautaire de Bangui, Avenue des Martyrs, Bangui, Central African Republic; 10 Complexe Pédiatrique de Bangui, Bangui, Central African Republic; 11 Centre de Traitement Ambulatoire de l’Hôpital Communautaire de Bangui, Bangui, Central African Republic; 12 Laboratoire de Virologie, Croix-Rousse Hospital, Lyon, France; 13 Department of Medical Informatics and Biostatistics, Iuliu Hatieganu University of Medicine and Pharmacy, Cluj-Napoca, Romania; University of Liverpool Institute of Infection and Global Health, UNITED KINGDOM

## Abstract

Hepatitis delta virus (HDV) increases morbidity in Hepatitis B virus (HBV)-infected patients. In the mid-eighties, an outbreak of HDV fulminant hepatitis (FH) in the Central African Republic (CAR) killed 88% of patients hospitalized in Bangui. We evaluated infections with HBV and HDV among students and pregnant women, 25 years after the fulminant hepatitis (FH) outbreak to determine (i) the prevalence of HBV and HDV infection in this population, (ii) the clinical risk factors for HBV and/or HDV infections, and (iii) to characterize and compare the strains from the FH outbreak in the 1980s to the 2010 HBV–HDV strains. We performed a cross sectional study with historical comparison on FH-stored samples (n = 179) from 159 patients and dried blood-spots from volunteer students and pregnant women groups (n = 2172). We analyzed risk factors potentially associated with HBV and HDV. Previous HBV infection (presence of anti-HBc) occurred in 345/1290 students (26.7%) and 186/870 pregnant women (21.4%)(*p = 0*.*005*), including 110 students (8.8%) and 71 pregnant women (8.2%), who were also HBsAg-positive (*p = 0*.*824*). HDV infection occurred more frequently in pregnant women (n = 13; 18.8%) than students (n = 6; 5.4%) (*p = 0*.*010*). Infection in childhood was probably the main HBV risk factor. The risk factors for HDV infection were age (*p = 0*.*040*), transfusion (*p = 0*.*039*), and a tendency for tattooing (*p = 0*.*055*) and absence of condom use (*p = 0*.*049*). HBV-E and HDV-1 were highly prevalent during both the FH outbreak and the 2010 screening project. For historical samples, due to storage conditions and despite several attempts, we could only obtain partial HDV amplification representing 25% of the full-length genome. The HDV-1 mid-eighties FH-strains did not form a specific clade and were affiliated to two different HDV-1 African subgenotypes, one of which also includes the 2010 HDV-1 strains. In the Central African Republic, these findings indicate a high prevalence of previous and current HBV-E and HDV-1 infections both in the mid-eighties fulminant hepatitis outbreak and among asymptomatic young adults in 2010, and reinforce the need for universal HBV vaccination and the prevention of HDV transmission among HBsAg-positive patients through blood or sexual routes.

## Author summary

In the Central African Republic (CAR), due to 20 years of conflict, the health system has been disorganized. This could contribute to maintenance of high transmission levels of Hepatitis B Virus (HBV) and its satellite Hepatitis Delta Virus (HDV). This work studies the evolution of both infections 25 years after a fulminant hepatitis (FH) outbreak occurring in the mid-1980s associated with HDV superinfection. In young asymptomatic adults, the results show that both HBV and HDV were still actively circulating in CAR in 2010. Indeed, more than one third of HBV-infected individuals were chronic HBV carriers. Furthermore, HDV infection could be spreading among 10% of them through blood and sexual transmission. The past FH outbreak and contemporary infections were both associated with heterogeneous HDV-1 strains, combined with HBV-E. Vaccination against HBV was uncommon among pregnant women and students, even among medical students. The study constitutes warning signals to help CAR health-care reconstruction and underlines the importance of HBV vaccination. The high level of HBV infection creates a background for HDV superinfection. Neonatal HBV vaccination is needed, together with vaccination of unprotected populations. Awareness of health authorities as well as the general public would help reduce HBV and HDV infections.

## Introduction

Hepatitis B virus (HBV) infection can be prevented by vaccination, which can obviate progression of chronic liver disease to cirrhosis, liver failure and hepatocellular carcinoma. However, owing to limited access to vaccination during the past 30 years in resource-poor countries, more than 2 billion people have been infected with HBV and approximately 257 million remain chronic carriers, especially those infected at birth or in early childhood [1].

Viral hepatitis can be associated with acute severe illness, sometimes leading to fulminant destruction of the liver parenchyma or to chronic inflammatory-based injury, resulting in liver remodeling and regeneration that contributes to fibrosis and early carcinogenic events. Both acute co-infection with HBV and hepatitis D virus (HDV) and superinfection of chronic HBV carriers by HDV can aggravate these clinical spectra [2,3], sometimes leading to fulminant hepatitis (FH) at higher rates than in HBV monoinfection. In the early years after HDV discovery, HDV co- and super-infection profiles accounted respectively for 58% and 42% of delta-associated FH in Western Europe [4]. Furthermore, liver fibrosis accelerates in chronic HBV and HDV infections and HDV may have a role in carcinogenesis [5, 6]. A recent meta-analysis studying the prevalence of HDV infection in Sub-Saharan countries indicated that in countries surrounding CAR, such as Cameroon, anti-HD Ab prevalence was up to 14% of HBsAg-positive individuals from a national survey [7]. Furthermore, in the Central African Republic (CAR) among patients followed up in hepatology clinics, the anti-HD prevalence reached 50% and this prevalence was 18% in patients with HCC [7].

Although HDV was identified only 40 years ago [3], it has probably been present in humans for a long time. The virus is ubiquitous, and the prevalence of HDV infection among HBsAg-carriers may vary from 0.5% to 40%, depending of the geographical area studied [2]. A global estimation of HDV-infected population suggests that 10–20 million HBV-infected people have encountered HDV. The virus has high genetic variability, especially in Africa, Amazonia and Asia [8,9]. Currently, the *Deltavirus* genus comprises eight major clades, or genotypes, labelled HDV-1 to HDV-8 [9,10]. HDV-1 is ubiquitous while HDV-2 and HDV-4 in Asia and HDV3 in Amazonia are geographically restricted. HDV-5–HDV-8 have a clear African origin but may be found elsewhere, due to slave trading and human migration [9,11]. While HBV is a DNA virus with a reverse transcriptase targeted by antiviral drugs, HDV RNA replicates by hijacking the cellular RNA polymerase II. Therefore, chronic hepatitis due to HBV–HDV is the most difficult-to-treat of all severe virally-induced liver diseases [12–14]. Even though therapeutics are available for chronic HBV or HCV infections, about 15 million HBV–HDV-infected patients worldwide await efficient therapy. Alpha-Interferon or Pegylated-IFN that are currently the sole anti-HDV authorized drugs, may be of limited efficacy in less than one third of infected patients. Furthermore, addition of Adefovir or Tenofovir or Entecavir to Pegylated-IFN does not improve the sustained response rate among HDV-1-infected patients, underlining the importance for developing new approaches [12,14]. Indeed, new strategies, such as viral entry competitors, farnesyl transferase inhibitors and nucleic acid polymers are nowadays promising, even if they are still upstream from a wide clinical use [14].

The severity of HBV–HDV infections could be due to particular evolutionary and/or geographical combinations of HBV–HDV genotypes, and some HBV–HDV pairs may be more pathogenic than others [15,16]. In the mid-1980s, cases of severe acute liver failure in Bangui, Central African Republic (CAR), were associated with an HDV outbreak. At least 124 cases of FH occurred among 154 cases of jaundice at Bangui University Hospital, and 88% of the patients died [17]. The evolution of HDV has not been studied since the outbreak, although the high prevalence of HBV infection [18] and the global instability in CAR constitute a potential background for the spread of HDV that may lead to severe chronic liver disease and increased risk of dying from liver failure. Furthermore, although a national HBV preventive program is still conducted, the civil war in the country might have maintained high levels of HBV and HDV transmission through injuries, blood and sexual contacts, mother-to-child transmission and even unsafe blood transfusion. Indeed, viral hepatitis infections, especially hepatitis B, are likely to continue to be a major problem in the near future in CAR. Therefore, it seems crucial to reappraise the level of HBV and HDV infection in young CAR patients to specify the level of unprotected persons that may benefit from catch-up vaccination, if they are free of infection, and those that might be included in future therapy protocols at a time where the World Health Organisation reflexion encourages specific hepatitis funding among infectious diseases global health strategies.

The aims of our study were (i) to determine the prevalence of HBV and HDV infection in young asymptomatic students and pregnant women born at the end or after the end of the FH outbreak, (ii) to determine possible clinical risk factors for HBV and/or HDV infections in this population and (iii) to characterize and compare the strains from the FH outbreak in the 1980s to 2010 HBV–HDV strains.

## Methods

We performed a cross sectional study with historical comparison.

### Prospective study population

Two cohorts, volunteer students (n = 1298) and pregnant women (n = 874), were prospectively sampled between January and December 2010 (S1 Method and S1 Table). Based on volunteering, students came from almost all public and private high schools in Bangui, the capital of CAR, and all different departments of the University of Bangui (S2 Table). Objectives of the study were carefully explained to high-school directors and to the university general secretary in order to obtain authorisation. The investigators then organized information meetings in each secondary school and faculty before distributing the information notice. Written consent forms were obtained from those who freely accepted to participate to the study. Therefore, this recruitment should be considered as passive. Interested students filled the questionnaire anonymously. Recruitment of pregnant women (PW) was performed in all public health structures (n = 6) with maternity wards in Bangui. In these places, medical staffs, including qualified midwives involved in the Mother-to-Child HIV transmission prevention (MCTP), were responsible for sampling. PW coming for prenatal consultation were sampled (dried blood spot) after an initial pre-test counselling. All consecutive pregnant women that accepted to participate to the study signed the consent form and answered the epidemiological questionnaire. Those who had an HBsAg positive result were then followed at the 2 reference public hospitals: “Communautaire” and “l’Amitié”, under the supervision of one of us (AS). No recruitment occurred in private clinics. In all, 874 consecutive pregnant women agreeing to participate were included. The information asked for all participants included gender, age, place of living in Bangui, occupation, marital status, personal viral hepatitis history and previous HBV vaccination. Risk factors investigated were surgery, dental extraction, blood transfusion, IVDU, tattooing, use of sharp tool, alcohol intake and sexual risk factors including ancient and actual sexual partners and use of condom (S1 Method: French-to-English translated questionnaire).

### Ethical approval

The study protocol was approved by the Scientific Committee of the Faculty of Medicine (CSVPRS, 12/2009; University of Bangui, CAR) and by a French ethical committee (CCPPRB, CPP 09024/2009; Saint-Germain-en-Laye, France) under ANRS guidelines. Written informed consent was obtained from all participants. For subjects under 18, parental consent was obtained. Each participant or parent was individually informed of the results of the serological tests.

### Samples

*1984–1987 outbreak*: Archived samples had been conserved in a cold room (–20°C) in Lyon, France, since the FH outbreak [17]. For HBsAg serological studies, we used 179 serum samples from 159 individuals, including patients and close relatives or health-care workers (HCWs), sampled at the same time. Because of insufficient volumes, we conducted molecular analyses for only 133 samples from FH patients and close relatives or HCWs. However, we could not distinguish between these categories because the identifiers were no longer available.

*Specimen collection in 2010*: Dried blood spots (n = 6/individual) were prepared as described previously [18]. Filter papers were sealed and stored at the Institut Pasteur de Bangui at –20°C with desiccant until serological assay. To ensure enough sample for molecular analyses of HBV and HDV, 10 mL of total blood were taken from the HBsAg-positive volunteer students (*see below*) and conserved at –80°C.

### Serological assays

Dried blood spots were examined as described previously [18]. All three markers HBsAg, anti-HBc and anti-HBs tests (Abbott-Murex, United Kingdom) were performed according to the manufacturers’ instructions in 2160 individuals’samples. HBsAg-positive results were confirmed in the Murex HBsAg Confirmatory test, Version 3. HBsAg-positive samples from the 1985 (n = 84) and 2010 (n = 181) serum collections were screened for HDV with the anti-HDV total antibody test and HDAg detection tests (diaSorin, Italy).

### HBV and HDV viral loads and sequence analysis

From the 114 HBsAg-positive students contacted, 82 individuals agreed to give serum samples for molecular analyses, including all HDV-infected students. Studies were performed at the University Paris 13, Bobigny, France, as previously described [19,20].

### Archived sample extraction and amplification

To avoid contamination with recent samples, archived samples were extracted and amplified in a separate laboratory in Lyon, France. Initially, Macherey-Nagel extraction procedures were used for 40 samples using RNA and DNA extraction kits to explore both HBV and HDV genotypes, respectively. For subsequent samples, due to the conservation conditions, all efforts were focused on HDV RNA detection and characterization. Total nucleic acids were extracted by an automated procedure (VERSANT^®^ kPCR, Siemens Healthcare). Control water extraction was included for every 10 samples. A 400 base pair HDV fragment was amplified according to reference [9]. (dx.doi.org/10.17504/protocols.io.mtdc6i6) with HiFi Kapa polymerase (KapaBiosystems). This RT-PCR procedure has been shown to be of higher sensitivity than the RT-qPCR approach [21] and was chosen to minimize the risk of underestimation of the results because of the sub-optimal conservation conditions of the historic samples (-20°C during 25 years). To amplify the full-length L-HD gene, we designed a 5’ primer corresponding to the seven first codons of HDV-1 sequences. For HBV genotyping, *PreS1* and *PreC-C* genes were amplified according to [19] (dx.doi.org/10.17504/protocols.io.m8jc9un and dx.doi.org/10.17504/protocols.io.mixc4fn, respectively). Unfortunately, despite many attempts, full-length sequence analysis of mid-eighties FH-associated strains was unsuccessful. Sequence analysis and phylogenetic studies were conducted with different models and Bayesian reconstruction, as described previously [9,10,20].

### Statistical analysis

Sample size was calculated with the following formula taking into account potential cluster effects:

N_0_ = Dz^2^pq/i^2^ where N_0_ is the sample size, p the expected HBV prevalence, q = 1-p, D the cluster effect (set at 1), and i the acceptable precision (set at 2%). Considering a theoretical HBsAg prevalence among students of 15% [18], the calculated sample size is 1257 (z = 1.96). For pregnant women, the required sample size based on a theoretical prevalence of 13% is 1087. Results are presented as numbers and percentages for qualitative variables, and as means +/- standard deviations (SD) for quantitative variables.

Pearson Chi-squared or, when necessary, Fisher exact tests were used to compare categorical data between the different groups. For continuous data, Student’s t test was used. Multivariate analysis using logistic regression was performed to identify factors potentially associated with HDV. A p value <0.05 was considered statistically significant. Statistical analyses were performed using SPSS for Windows Version 19.0 (IBM Corp., Armonk, NY, USA).

## Results

### HDV serological assay results in the historical cohort

Among the 159 individuals, identifiers of their status such as health care worker were no longer available, although four family clusters (n = 6, 6, 2, 2 members, respectively) could be distinguished. Therefore, to study hepatitis delta markers, we retrospectively focused on samples having an HBsAg positive result (n = 94 patients). For 84 of them (89.4%), both HDAg and total anti-HD Ab were tested using commercial ELISA tests. Results are described in Table 1. HDAg and anti-HD total Ab tests were both positive in 4/84 samples, HDAg was the only positive delta marker in 24/84 samples and anti-HD total Ab was the only marker in 23/84 samples, making 51/84 (60.7%) samples that had at least one positive HDV serological marker. These results are to be regarded in the context of the fulminant acute HDV/HBV outbreak, at a time when anti-HD Abs could not be detectable in a large number of samples corresponding to Day1, Day2 or Day3 of the severe acute liver disease.

**Table 1:** Prevalence of HBV and HDV markers in 2172 young asymptomatic adults (students and pregnant women) in Bangui (CAR), in year 2010. Historical cohort (n = 159) corresponds to -20°C cryopreserved serum from a hepatitis delta fulminant outbreak and control samples collected in the mid-eighties. Data are mean (SD), or n (%) unless otherwise stated. ND, not determined.

Prospective cohort (n = 2172)	Students (n = 1298)	Pregnant women (n = 874)	Total (n = 2172)	*P*^1^	Historical cohort (n = 159)
Age (mean ± SD) (n = 2170)	21.9 ± 4.0	25.0 ± 5.7	23.1 ± 5.0	<0.001	ND
Sex (F/M) (n = 2170)	448/848	874/0	1322/848	<0.001	ND
Previous vaccination (n = 2165)	20 (1.5%)	23 (2.6%)	43 (2.0%)	0.074	ND
HBsAg positive^2^ (n = 2160)	110 (8.5%)	71 (8.2%)	181 (8.4%)	0.824	94/154 (61.3%)
Anti-HBc IgG positive (n = 2160)	345 (26.7%)	186 (21.4%)	531 (24.6%)	0.005	ND
Anti-HBc IgG positive among HBsAg negative (n = 1979)	235 (19.9%)	115 (14.4%)	350 (17.7%)	0.002	ND
HBsAg and/or anti-HBc IgG positive (n = 2160)	345 (26.7%)	186 (21.4%)	531 (24.6%)	0.005	ND
Anti-HDV Ab positive^3^	5/110 (4.5%)	13/69 (18.8%)	18/179 (10.0%)	0.004	27/84^4^ (32.1%)
HDAg positive^3^	1/110 (0.9%)	0/69 (0.0%)	1/179 (0.5%)	1.000	28/84^4^ (33.3%)
HDV infection^3^^,^^5^	6/110 (5.4%)	13/69 (18.8%)	19/179 (10.6%)	0.010	51/84^5^ (60.7%)

^1^Comparison Students versus Pregnant Women

^2^Five students among 2165 having HBsAg positive results did not have anti-HBc testing and were discarded from this analysis

^3^Among 179 recent HBsAg-confirmed positive results

^4^Due to insufficient serum volumes HDV serological tests were performed on 84/94 HBsAg-positive historical samples. Four patients had both HDAg and anti-HD Ab positive results

^5^Considering positive Anti-HDV Ab and/or positive HDAg results

### Prospective 2010 survey

HBV infection was highly prevalent in this highly-educated young asymptomatic population. When anti-HBc was used as a marker of previous HBV infection among 2160 individuals, 345 students (26.7%) and 186 pregnant women (21.4%) had encountered HBV at some time (Table 1). HBsAg was present in samples from 110 students (8.5%) and 71 pregnant women (8.2%) (*p = 0*.*824*) (Table 1). Of the 531 subjects positive for anti-HBc, 181 (110 + 71) had HBsAg detectable in their serum, indicating a high chronicity rate of 34.1%. As the risk of becoming a chronic carrier is higher in young children than in adolescents or adults and because no significant known adult risk factors of transmission emerged when comparing HBV-positive *versus* HBV-negative individuals (Table 2 and S3 Table and S4 Table), this result may indicate that possible major routes for HBV infection were (i) being born from an HBsAg-positive mother or (ii) being infected in the very first years of life. To avoid confounding factors, each cohort was also studied specifically comparing the HBV-non infected versus the HBV-infected individuals (S5 Table and S6 Table). Age was slightly significantly associated to HBV previous or current infection in students, whereas previous hepatitis and alcohol intake were identified as risk factors in the pregnant women group.

**Table 2:** Comparison of HBV status among young asymptomatic adults (students and pregnant women) in Bangui, Central Africa Republic (CAR) in 2010.

Anti-HBc antibodies AND/OR HBsAg			Negative (n = 1631)	Positive (n = 531)	*p*
Group	Students (n = 1296)		947 (73.1%)	349 (26.9%)	0.002
	Pregnant Women (n = 866)		684 (79.0%)	182 (21.0%)	
Age (years; mean ± SD)			23.1 ± 5.0	23.3 ± 5.0	0.456
Female:male			997:634	317:214	0.558
Marital status (n/%)					0.156
	Single		1378 (84.5%)	454 (85.5%)	
	Live-in partnership		133 (8.2%)	50 (9.4%)	
	Married (monogamous)		89 (5.5%%)	18 (3.4%)	
	Married (polygamous)		29 (1.2%)	7(1.3%)	
	Widowed		2 (0.1%)	2 (0.4%)	
District Address in Bangui			See details in S3 Table		0.769
Occupation			See details in S4 Table		0.600
CAR nationality			1620 (99.3%)	526 (99.1%)	0.561
Risk factors	Previous viral hepatitis (n = 2045)		27 (1.8%)	15 (3.0%)	0.094
	Previous icterus (n = 2148)		132 (8.2%)	55 (10.4%)	0.112
	Surgery (n = 2162)		126 (7.7%)	44 (8.3%)	0.677
	Dental extraction (n = 2155)		491 (30.2%)	138 (26.1%)	0.080
	Blood transfusion (n = 2152)		75 (4.6%)	26 (4.9%)	0.874
	Tatoo (n = 2162)		102 (6.3%)	35 (6.6%)	0.782
	Intravenous drug user (n = 2162)		8 (0.5%)	3 (0.6%)	0.531
	Sharp-edged tool use (n = 2162)		851 (52.2%)	270 (50.8%)	0.594
	Alcohol (n = 2162)		784 (48.1%)	264 (49.7%)	0.509
	Multiple partners before (n = 2162)		579 (35.5%)	201 (37.9%)	0.327
	Multiple partners in 2010 (n = 2162)		98 (6.0%)	35 (6.6%)	0.627
	Use of condom-(n = 2075)	Always	414 (26.4%)	147 (29.0%)	0.045
		sometimes	375 (23.9%)	140 (27.6%)	
		never	779 (49.7%)	220 (43.4%)	
Previous HBV vaccination	(n = 2156)		31 (1.9%)	11 (2.1%)	0.801

HDAg and total anti-HD (IgM/IgG) were studied in sera from 110/110 (100%) HBsAg-positive students and 69/71 (97.2%) HBsAg-positive pregnant women. Five students and 13 pregnant women had anti-HD antibodies in their serum, and one student, without detectable anti-HD, had a positive HDAg result thus considered to have acute hepatitis D. Therefore, the prevalence of HDV infection was 6/113 students (5.4%) and 13/69 pregnant women (18.8%). Indeed, whereas HBsAg prevalence was similar between the two groups, HDV prevalence was significantly higher among pregnant women than students (Table 1, *p = 0*.*010*). The possible contributing factors for HDV acquisition were a higher mean age (*p<0*.*001*), higher rates of previous blood transfusion (*p< 0*.*039*) and tattooing (*p<0*.*055*) and a lower rate of condom use (*p = 0*.*049*) (Table 3). Concerning this last point, we could not rule out a confounding factor as pregnant women obviously did not always have condom-protected sex and were significantly more frequently infected with HDV than students. On the other hand, we cannot exclude age as an HDV increased risk factor, as this was found, although non significantly, in each cohort considered independently (S7 Table and S8 Table). Because the end of the fulminant hepatitis outbreak occurred late 1987, older students and pregnant women would have been born when HDV transmission risk was still present.

**Table 3:** Comparison of HDV status among 182 HBsAg-positive young asymptomatic adults (students and pregnant women) in Bangui, Central Africa Republic (CAR) in 2010.

HDV (Anti-HD AND/OR HDAg)		Negative (n = 163)	Positive (n = 19)	*p*
Group	Students (n = 113)	107 (94.7%)	6 (5.3%)	0.004
	Pregnant Women (n = 69)	56 (81.2%)	13 (18.8%)	
Age (years; mean) [SD]		23.30 ± 5.09	25.89 ± 5.76	0.040
Female:male		103/60	17/2	0.022
Marital status (n/%)				0.736
	Single	139 (85.3%)	16 (84.2%)	
	Live-in partnership	15 (9.2%)	2 (10.5%)	
	Married (monogamous)	5 (3.1%)/	1 (5.3%)/	
	Married (polygamous)	2 (1.2%)	0 (0.0%)	
	Widowed	2 (1.2%)	0 (0.0%)	
District Address in Bangui		See S3 Table		0.165
Occupation		See S4 Table		0.067
CAR nationality		160 (98.2%)	18 (94.7%)	0.359
Risk factors	Previous viral hepatitis (n = 174)	5 (2.6%)	2 (10.5%)	0.170
	Previous icterus (n = 182)	15 (8.2%)	2 (10.5%)	0.553
	Surgery (n = 182)	12 (6.6%)	1 (5.3%)	0.596
	Dental extraction (n = 182)	40 (22.0%)	8 (42.1%)	0.100
	Blood transfusion (n = 182)	5 (2.7%)	3 (15.8%)	0.039
	Tatoo (n = 182)	11 (6.0%)	4 (21.1%)	0.055
	Intravenous drug user (n = 182)	1 (0.5%)	0 (0.0%)	0.896
	Sharp-edged tool use (n = 182)	85 (46.7%)	8 (42.1%)	0.407
	Alcohol (n = 182)	81 (44.5%)	10 (52.6%)	0.808
	Multiple partners before (n = 182)	67 (36.8%)	6 (31.6%)	0.423
	Multiple partners in 2010 (n = 182)	13 (7.1%)	1 (5.3%)	0.557
	Use of condom:- always	43 (27.0%)	5 (26.3%)	0.049
	(n = 178) - sometimes	47 (29.6%)	1 (5.3%)	
	- never	69 (43.4%)	13 (68.4%)	
Previous HBV vaccination	(n = 181)	0 (0.0%)	1 (5.3%)	0.105

### HBV DNA amplification and sequencing

Nucleic acid testing for HBV sequences was achievable for 40 historical samples with sufficient residual volume, and the *Pre-C/C* region of HBV could be amplified and sequenced from eight samples. Seven sequences were of HBV genotype E and one of genotype A2 (S1A Fig). For the contemporary survey, of the 110 HBs-Ag positive students, 82 (71.9%) agreed to return for medical examination and virus analyses. The HBV DNA viral loads were quantified, except for two samples, in which amplification failed. In the 80 remaining samples, HBV-DNA was undetectable in seven (8.75%), at a low level (< 3.3 log10 IU/mL) in 33 (41.25%) at a medium level (> 3.3 < 8.3 log10 IU/mL) in 18 (22.5%) and at a high level (> 8.23 log10 IU/mL) in 22 (27.5%), indicating that half of the asymptomatic students had moderate-to-high viral replication. Amplification of the *Pre-S1* coding region of the HBV genome resulted in 50 interpretable HBV sequences that were submitted to Bayesian analyses with reference sequences (S2A Fig). Tree reconstruction indicated that most of CAR2010 were of HBV genotype E (n = 48) whereas 2 CAR2010 strains corresponded to HBV_DE recombinants (S2A Fig and S1B Fig and S2B Fig). In addition, we then aimed to amplify the *PreC/C* from 2010 CAR HBV strains and obtained 35 sequences all affiliated to HBV genotype E (S1B Fig). We also tried to amplify the *Pre-S1* coding region from the 40 HBsAg positive historical samples for which DNA had been extracted, focusing on HBsAg-positive ones. We could obtain 5 *Pre-S1* sequences corresponding to 3 patients with fulminant hepatitis (HF2, HF19 and HF27). Tree reconstruction, focused on HBV D and E genotypes, indicates that all three FH-associated HBV strains corresponded to HBV_E genotype and one of them could cluster with one CAR2010 HBV strain (S2B Fig).

### HDV RNA amplification and sequencing

**Fig 1:**
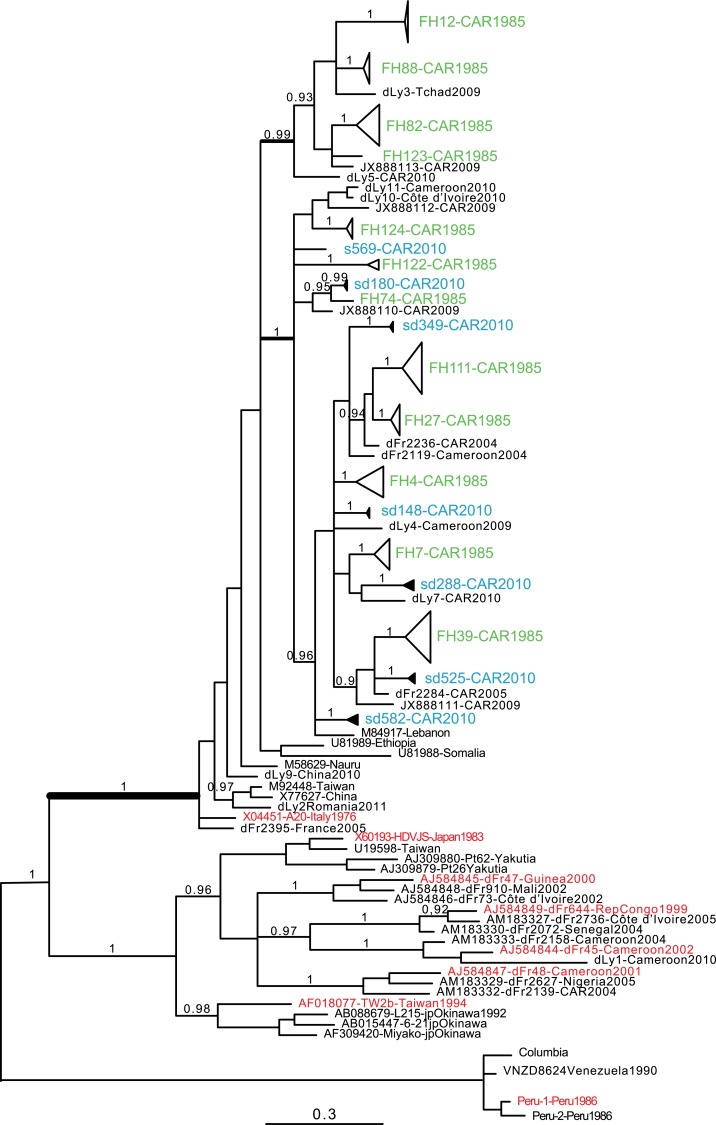
Phylogenetic analysis of HDV partial genome (*400 bp*) comparing mid-eighties delta fulminant hepatitis clones (FH1985) to HDV direct sequences (CAR2010) sampled in 2010 from serum (s) and dry blood spot (d) among asymptomatic young adults in Bangui. We aligned 45 cloned sequences from the 12 FH strains obtained from the historical cohort (labelled ‘1985’ in green) and the 6 strains obtained from asymptomatic students in 2010 represented in duplicate from serum (s) and dried blood (d) (labelled ‘2010’ in blue). We also included 1 strain from a hospitalized case of acute HDV hepatitis in Bangui in 2010 (sd525-CAR2010) and HDV-1 sequences from 9 African samples characterized in Bobigny (dFr) or Lyon (dLy), France, in addition to CAR HDV sequences published by Andernach and coworkers [22], sampled in 2009. Further comparison included HDV strains from various parts of the world and genotype-reference prototypes (labelled in red). Bayesian analyses (10M generations) gave the consensus tree represented in Fig 1, after discarding 25% of trees from early topology exploration. Branch values indicate posterior probabilities >0.9. Interestingly, the fulminant 1985 and asymptomatic student 2010 strains are all affiliated to HDV-1 with a 100% posterior probability value (thick branch) and the clade topology do not distinguish the mid-eighty strains from the 2010 strains.

Among 88 of the 94 HBsAg-positive historical samples (Table 1) that were available for HDV molecular studies, a 400-bp target encompassing the 3’ coding region of the *LHD* gene was successfully amplified, cloned and sequenced from 12 HDV-infected patients. Full-length *LHD* gene amplification from historical samples was successful in only three cases, although several clones were sequenced from each. For the 2010 samples from the 6 HDV-positive students, the 400-bp target could be amplified in five cases from both serum and dried blood spot and from serum only in one case. All sequences from historical and contemporary samples were unambiguously belonging to the HDV-1 genotype (Fig 1). A Bayesian reconstruction consensus tree, obtained using CAR historical and contemporary sample sequences from this study, African (Cameroon, CAR, Chad, Côte d'Ivoire) sequences (S9 Table), and sequences retrieved from databases, is displayed in Fig 1. The database sequences included the eight HDV genotype-prototype sequences (shown in red in Fig 1) and other HDV-1 CAR sequences characterized in 2009 [22].

**Fig 2:**
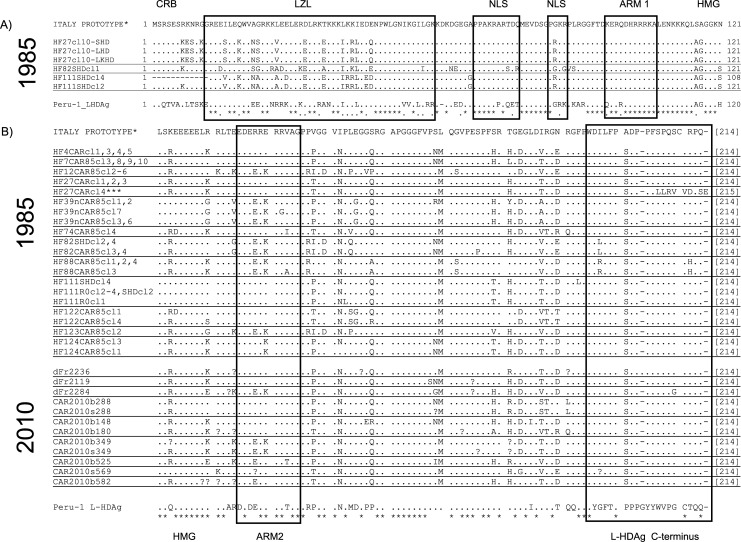
Alignment of the L-HDAg amino acid deduced sequences from fulminant-associated isolates from the historical cohort (1985) and from young asymptomatic HDV-infected students (2010). The alignment is compared together with L-HDAg sequences from HDV-1 prototype (Italy, Accession Number:X04451) and HDV-3 prototype (Peru-1, Accession Number:L22063), representing the prototypes of two HDV genotypes associated with fulminant hepatitis outbreaks. Dots represent the same amino acid as in the Italy prototype and question mark ambiguities. A: Full-length coding sequences (214 codons) were obtained for 3 FH isolates (FH27, FH88, FH123). B: COOH terminal part of L-HDAg of the corresponding HDV 1985 (45 FH clones) associated with fulminant hepatitis and 2010 (seven) sequences associated to asymptomatic infections. Note that all the African sequences have the A202S mutation and that FH27 clone 4 has a frameshift mutation of the carboxy-terminal end of L-HDAg (ORF-K), leading to the disappearance of the farnesylation CXXX box.

The topology of the tree clearly indicates that the FH viral sequences do not form a specific historical clade. Thus, all HDV sequences from the HF-mid-eighties strains did not derive from a common ancestor in a single phylum, unrelated to the recent HDV sequences retrieved from asymptomatic students. Instead, one large clade intercalates historical and recent sequences from our study in CAR, plus other Central and West African HDV-1 strains, and sequences from Ethiopia, Somalia and Lebanon characterised previously. Inside this clade, there is one strong sustained branch (posterior probability (*pp*) = 0.99) that includes four FH strains (FH12, FH82, FH88 and FH123) and sequences from strains that infect patients in CAR and Chad. All the other FH strain sequences cluster (*pp* = 1) with isolates from patients originating from CAR, Cameroon, Côte d’Ivoire and Lebanon. Among HDV-1 strains, recent full-length HDV genome analyses have raised the possibility of the existence of at least 4 HDV subgenotypes [23]. Even if we could only obtain partial sequences due to conservation conditions, we reanalysed the HDV sequences and conclude that FH12, FH82 FH88 and FH123 were clearly affiliated to the suggested HDV-1a (Ancestral African) subgenotype. In contrast, the other ancient and recent trains clustered together with the Lebanon strain suggesting that they corresponded to HDV-1b subgenotype stains. Although a formal analysis should have included full- length sequences, these results reinforce the fact that the fulminant hepatitis outbreak in the mid-eighties was linked to very different HDV-1 strains. Translation of the coding region for the C-terminus of HDAg showed a serine residue at position 202 in all sequences (Fig 2), corroborating an African HDV-1 affiliation [20]. FH27 isolate clone 4 sequence had a nucleotide deletion leading to a frameshift mutation in the L-HDAg sequence that abrogates the farnesylation signal. Otherwise, the comparison of L-HDAg amino acid sequences from historical FH samples with recent samples revealed no specific FH-associated signature.

## Discussion

To study the persistence of hepatitis B and hepatitis Delta viruses twenty-five years after a major HDV and HBV FH outbreak in an equatorial city in Africa, we conducted a prospective search among young healthy adults in 2010. The study was not designed to specify HBV and HDV prevalence in extensive Bangui populations, but was focused on individuals born at or after the end of the epidemic outbreak, to assess viral long-term continuance. After patient inclusion, questionnaire filling and screening, further actions included volunteer-based HBV vaccination and medical examination, for seronegative individuals and HBsAg-positive carriers, respectively.

An important observation is that both HBV and HDV are still actively circulating in CAR. Indeed, both students and pregnant women had a high prevalence of previous HBV infection and HBsAg-chronic carriage. In this survey, including a highly educated population, more than one third of HBV-infected individuals were chronic carriers. It has been estimated that 90% of newborns infected at or shortly after birth will become HBV carriers, although this rate decreases with age, until less than 5–10% for adolescent or adult HBV-infections [24]. In this study, most of the students and pregnant women who were anti-HBc-positive had probably been infected at an early age, indicating the importance of sustaining an anti-HBV vaccine strategy for newborns in CAR. Furthermore, HDV could be amplified from all six students with anti-HD or HDAg, indicating continuing HDV replication and an active reservoir that also could have been prevented by early anti-HBV vaccination. Indeed, an important issue corresponds to the fact that a very low prevalence of earlier anti-HBV vaccination (<2%) was observed and that a failure for students to participate for a *de novo* free anti-HBV vaccination was evidenced, both indicative of HBV (and HDV) potential acquisition in adulthood.

HDV infection was still active in 2010 cohorts, however, due to our study design focused on asymptomatic young adults, no fulminant hepatitis was observed in 2010 infections. By comparison, a study conducted in the Democratic Republic of Congo revealed than HBV-HDV could be found in 26.1% of acute febrile jaundice samples from a national yellow fever survey from 2003 to 2011 [25].

In CAR, HBV-E and HDV-1 were present in the 1980s and 2010 and no specific fulminant hepatitis-associated HDV strain pattern was clearly evidenced comparing 1980s to 2010 strains. During the FH outbreak in Bangui in the mid-1980s, the disease was associated with specific pathological lesions (spongiocyte-associated hepatitis) involving lympho-plasmocytoid infiltrates, lesions of eosinophilic necrosis and massive macro- or micro-vacuolar steatosis, giving cells a particular "morula-like" type and resulting in a high mortality rate [17,26]. Such strain-associated lesions could be propagated in the woodchuck model [26]. Similar lesions have been described in South American FH outbreaks [27,28] that were linked to HDV-3, usually associated with HBV-F, although HBV-A and HBV-D have also been implicated in Amazonia [29,30]. In Bangui, the outbreak involved HDV-1, here associated with HBV-E. This particular histopathological type of FH, associated with high mortality, was therefore not due to a single HBV–HDV genotypic association. During the outbreak, it may be hypothesised that a recent combination of different HDV-1 subgenotypes with the Twentieth Century-introduced African HBV-E helper, rather than HBV-A or HBV-D, could have resulted in a severe disease. Such an explanation was suggested in a study in Taiwan, where the spread of HDV-1 to the Asian HBV-B and HBV-C genotypes resulted in more FH than the indigenous HDV-2 [31]. Alternatively, there may be in equatorial rainforests some as yet unrecognised cofactor that might increase the number of cases of fatal acute liver failure observed in both CAR and Amazonia [25,32].

In this study, the potential role of specific HDV-1 strains or its HDAg protein in the pathological lesions was looked for. We found no evidence of a specific HDV-1 fulminant associated clade. Indeed, within the limits of the sequences obtained, the 1985 HDV-1 FH strains were indistinguishable from asymptomatic-associated 2010 HDV-1 strains. Due to conservation conditions since the outbreak, we were unfortunately unable to amplify full-length FH-associated HDV genomes, even using many different approaches. This obviously cannot rule out some specific virulence factors that could have been deduced from comparison of full-length genomes between historical fulminant strains and recent asymptomatic-associated strains. The FH strains and their HDAg sequences intermingle with strains isolated from 2010 asymptomatic patients in CAR and with strains isolated elsewhere in Africa. The only exception corresponded to one HDV clone that displayed a frameshift mutation at the 3’ terminus of the *L-HDAg* gene that would eliminate the farnesylation signal of L-HDAg (Fig 2, *FH27 clone 4*). This may affect virion assembly and perhaps induces intracellular retention of viral component that may accumulate in infected cells. This variant was observed only once among 45 clones analysed from 12 FH-HDV strains. However, it can be hypothesised that mutants leading to intracellular retention may not easily egress from the infected-cell and might therefore be under-represented in the serum. In addition, they may be potentially cytopathic for the infected hepatocytes. For example, in HBV monoinfection of the human hepatocyte, retention of HBV-PreS1 mutants results in specific pathological lesions known as "ground-glass" hepatocytes [33].

It is disappointing that vaccination against HBV is so uncommon among pregnant women and students with a high education level, even among medical students. Neonatal vaccination (DTP-HepB-Hib) has been practised in CAR since 2008, but coverage is still not universal (http://www.gavi.org), in part due to civil unrest. At present, there is also no HBV immunotherapy nor immediate vaccination of neonates born outside hospitals, in the absence of qualified midwifes [1]. Indeed, modelisation has suggested that the proportion of new chronic cases arising from mother-to-child transmission may increase by up to 50% in 2030 [34]. Neonatal anti-HBV vaccination is urgently needed, preferably including a dose as near as possible after birth. Ideally, vaccination of as yet unprotected young populations should also be sustained [1,34]. Meanwhile, at least 8% of the young population of Bangui is at risk for further HDV superinfection and related liver damage. The situation outside of the nation’s capital is probably worse as a recent evaluation has estimated an HBsAg rural prevalence of 11.65% in CAR [35]. The results of this study should clearly contribute to sensitise the health authorities to consider HBV and HDV infections as a major health challenge in the Central African Republic, and could lead to specific surveys, screening and medial actions among populations at risk of HBV acquisition and/or transmission.

## Supporting information

S1 Checklist STROBE statement. (DOC)

S1 Table Age, marital status, district address in Bangui, and blood borne and sexual risk factors in students and pregnant women in Central Africa Republic (CAR) in year 2010. (DOC)

S2 Table Students repartition among high schools and University departments. This list corresponds to almost all different high educational entities in Bangui, Central African Republic.
(DOC)

S3 Table Place of living of the studied cohort in the different districts (1–8) of Bangui capital and Bimbo suburbs district in function of HBV and HDV status. The gray scale indicates the economical status of the district.
(DOC)

S4 Table Occupation of subjects from the 2 cohorts (student and pregnant women) in function of HBV and HDV status. (DOC)

S5 Table Comparison of age, marital status, and blood borne and sexual risk factors in students infected or non-infected by HBV (HBV positive versus negative) in CAR in year 2010. (DOCX)

S6 Table Comparison of age, marital status, and blood borne and sexual risk factors in pregnant women infected or non-infected by HBV (HBV positive *versus* negative) in CAR in year 2010. (DOCX)

S7 Table Comparison of age, marital status, and blood borne and sexual risk factors in students infected or non-infected by HDV (HDAg ± anti-HD Abs positive *versus* negative) in CAR in year 2010. (DOCX)

S8 Table Comparison of age, marital status, and blood borne and sexual risk factors in pregnant women infected or non-infected by HDV (HDAg ± anti-HD Abs positive *versus* negative) in CAR in year 2010. (DOCX)

S9 Table HDV sequences from this study
Accession Summary -----------------
Type                 Accession
Unique Name Study PRJEB24597
ena-STUDY-Research Cancer Center of Lyon-24-01-2018-10:05:53:847–48
European Nucleotide Archive (ENA).
(DOC)

S1 Fig *A*: Consensus tree obtained from the *PreC-C* region of the HBV genome (nt1814–nt2495) of reference sequence from Galibert et *al*. (Nature. 1979). HBV DNA was amplified from DNA extracted from the 40 initial ancestral samples with Macherey Nagel extraction procedures. Amplification yielded positive results in 8 samples, and DNA was directly sequenced from both strands. Corresponding sequences were submitted to alignment by Clustal Omega (http/EBI), with 57 references sequences corresponding to pure HBV genotypes and subgenotypes enriched with HBV-E and DE recombinants [19] and submitted to Bayesian analyses.
*B*: Consensus tree obtained from the *PreC-C* region of the HBV genome (nt1821-nt2066), focused on HBV A, D and E genotypes. HBV DNA was amplified and sequenced from 2010 samples (35 HBV *PreC-C* sequences). Alignment was manually corrected and submitted to distance models and Bayesian inference. Note that phylogenetic information of the *PreC-C* region could not well differentiate HBV_D and HBV_E genotypes.
(EPS)

S2 Fig *A*: Consensus tree obtained from *PreS1* region analysis of the HBV genome (nt 2817–nt81) of reference sequence from Galibert *et al*. (Nature. 1979) obtained from 49 isolates from asymptomatic HBV-infected students sampled in Bangui in 2010. *PreS1* sequences were obtained by hemi-nested PCR as previously described [20]. Bayesian analyses were conducted on 3.10E6 generation; 25% of initial searches was discarded. Note that 48 isolates belong to the E clade and that using the *PreS1* region genotypes D, E and G were affiliated together with a 100% posterior probability value at the original common branch, whereas each of the 2 clades E and D had a *pp* of only 50%. This may be related to the sequences located in between, including FN594771, known to be D/E recombinant strains.
*B*: Consensus tree obtained from *PreS1* region analysis of the HBV genome (nt2838-nt58), focused on HBV_D and HBV_E genotypes. Note that historic sequences FH2, FH19 and FH27 were affiliated to HBV_E genotype strains, one of them clustering with a 2010 CAR HBV sequences s506preS1.
(EPS)

S1 Method French to English translated questionnaire. (DOCX)
