# Supplementary material for: Hepatitis B and hepatitis D virus infections in the Central African Republic, twenty-five years after a fulminant hepatitis outbreak, indicate continuing spread in asymptomatic young adults
Source: PLoS Negl Trop Dis. 2018 Apr 26;12(4):e0006377. doi: 10.1371/journal.pntd.0006377 (PMC5940242; doi:10.1371/journal.pntd.0006377)
Supplement: S1 Checklist — (DOC) [file pntd.0006377.s001.doc]

S1 - STROBE Statement—checklist of items that should be included in reports of observational studies

Manuscript

*Hepatitis B and hepatitis D virus infections in the Central African Republic, twenty-five years after a fulminant hepatitis outbreak, indicate continuing spread in asymptomatic young adults*

|  | Item No | Recommendation |
| --- | --- | --- |
| **Title and abstract** | 1 | (*a*) Indicate the study’s design with a commonly used term in the title or the abstract:  *Abstract section, Paragraph 2* : *We performed a cross sectional study with historical comparison on FH-stored samples (n=179) from 159 patients and dried blood-spots from volunteer students and pregnant women groups (n=2172).* |
|  |  | (*b*) Provide in the abstract an informative and balanced summary of what was done and what was found: *see Abstract section and Author summary* |
| Introduction | | |
| Background/rationale | 2 | Explain the scientific background and rationale for the investigation being reported:  *Introduction section*  *Paragraph 1: HBV Background, Paragraph 2 HDV pathogenesis and prevalence in Africa, Paragraph 3 : mondial prevalence, genetic variability of HDV and rare therapeutic options, Paragraph 4 Historical Fulminant hepatitis outbreak I CAR* |
| Objectives | 3 | State specific objectives, including any prespecified hypotheses:  Introduction section, Paragraph 5 and Paragraph 6: “*The aims of our study were (i) to determine the prevalence of HBV and HDV infection in young asymptomatic students and pregnant women born at the end or after the end of the FH outbreak, (ii) to determine possible clinical risk factors for HBV and/or HDV infections in this population and (iii) to characterize and compare the strains from the FH outbreak in the 1980s to 2010 HBV–HDV strains.*” |
| Methods | | |
| Study design | 4 | Present key elements of study design early in the paper:  *Methods section first sentence* “*We performed a cross sectional study with historical comparison*” |
| Setting | 5 | Describe the setting, locations, and relevant dates, including periods of recruitment, exposure, follow-up, and data collection: *Methods section, paragraph 1, subheading: Prospective study population; paragraph 3, subheading: Samples* |
| Participants | 6 | (*a*) *Cross-sectional study*—Give the eligibility criteria, and the sources and methods of selection of participants: *Methods, subheadings Prospective study population; subheading: Samples; subheading: Statistical analyses* |
|  |  | (*b*) *Cohort study*—For matched studies, give matching criteria and number of exposed and unexposed; *see Table 1, Table 2 and Table 3 and supplementary tables 1-8* |
| Variables | 7 | Clearly define all outcomes, exposures, predictors, potential confounders, and effect modifiers. NA; *See potential confounders and effect modifiers in supplementary tables 5-8*  Give diagnostic criteria, if applicable: *Methods subheadings “Serological Assays” and* “*HBV and HDV viral loads and sequence analysis”* |
| Data sources/ measurement | 8* | For each variable of interest, give sources of data and details of methods of assessment (measurement). Describe comparability of assessment methods if there is more than one group *see Method paragraph 1 subheading: Prospective study population; Paragraph 4 Serological assays; Paragraph 5 HBV and HDV viral loads and sequence analysis; Paragraph 6 subheading: Statistical analyses and alsoSsupplementary method S1 - French-to-English translated Questionnaire* |
| Bias | 9 | Describe any efforts to address potential sources of bias: *See Methods: “Archived sample extraction and amplification” and Results “HBV amplification and sequencing” and “HDV amplification and sequencing”and supplementary tables 5-8* |
| Study size | 10 | Explain how the study size was arrived at *Methods “Statistical analyses*” |
| Quantitative variables | 11 | Explain how quantitative variables were handled in the analyses. If applicable, describe which groupings were chosen and why: *Results HBV amplification and sequencing* |
| Statistical methods | 12 | (*a*) Describe all statistical methods, including those used to control for confounding  *See Methods Paragraph 7: subheading Statistical Analyses and supplementary tables 5-8* |
|  |  | (*b*) Describe any methods used to examine subgroups and interactions: *See Methods Paragraph 7: subheading Statistical Analyses and Table 1, Table 2 and Table 3 and supplementary Tables 1-8* |
|  |  | (*c*) Explain how missing data were addressed *Abstract Paragraph 2 and Methods Paragraph 3: subheading samples and Paragraph 4: subheading Serological assays, Results Subheading Prospective 2010 Survey, paragraphs 1 and 2; Table 1, Discussion: Paragraph 4* |
|  |  | (*d)* *Cross-sectional study*—If applicable, describe analytical methods taking account of sampling strategy: *Method, paragraph 7: subheading “ Statistical analysis”* |
|  |  | (*e*) Describe any sensitivity analyses*; see Methods Paragraph 3: samples and paragraph 5: subheading “HBV and HDV viral load and sequence analysis” and paragraph 6: subheading “Archived samples extraction and amplidfication”.* |

| Results | | |
| --- | --- | --- |
| Participants | 13* | (a) Report numbers of individuals at each stage of study—eg numbers potentially eligible, examined for eligibility, confirmed eligible, included in the study, completing follow-up, and analysed: *See Method Paragraph 1: subheading “Prospective study population”, Paragraph 3: subheading “Samples”, Paragraph 4: subheading “Serological Assays” and Tables 1, 2 and 3 and supplementary tables 1-8* |
|  |  | (b) Give reasons for non-participation at each stage*: Methods paragraph 1: subheading “Prospective study population” Paragraph 4: subheading “Serological Assays” Paragraph 5: subheading HBV and HDV viral load analysis.* |
|  |  | (c) Consider use of a flow diagram *N/A* |
| Descriptive data | 14* | (a) Give characteristics of study participants (eg demographic, clinical, social) and information on exposures and potential confounders: *See Methods,paragraph 1: subheading “Prospective study population”, and supplementary table 3 and supplementary table 4 and supplementary tables 5-8* |
|  |  | (b) Indicate number of participants with missing data for each variable of interest: *indicated in all tables* |
|  |  | (c) *Cohort study*—Summarise follow-up time (eg, average and total amount) *see discussion Paragraph 2* |
| Outcome data | 15* | *Cohort study*—Report numbers of outcome events or summary measures over time N/A |
|  |  | *Case-control study—*Report numbers in each exposure category, or summary measures of exposure: *N/A* |
|  |  | *Cross-sectional study—*Report numbers of outcome events or summary measures N/A |
| Main results | 16 | (*a*) Give unadjusted estimates and, if applicable, confounder-adjusted estimates and their precision (eg, 95% confidence interval). Make clear which confounders were adjusted for and why they were included *See Results section, Paragraph 1: subheading “Prospective Study population, including Table 1, Table 2 and Table 3 and supplementary Tables 5-8* |
|  |  | (*b*) Report category boundaries when continuous variables were categorized *See Results: Paragraph 3: subheading “HBV DNA amplification and sequencing”* |
|  |  | (*c*) If relevant, consider translating estimates of relative risk into absolute risk for a meaningful time period N/A |
| Other analyses | 17 | Report other analyses done—eg analyses of subgroups and interactions, and sensitivity analyses: *see Results section Paragraph 1: Subheading “Prospective Study population and supplementary tables 1-8, Paragraph 3: subheading “HBV DNA amplification and sequencing” and paragraph 4 subheading “HDV RNA amplification and sequencing including Figure 1 and 2 and supplementary figures 1 and supplementary figures 2* |
| Discussion | | |
| Key results | 18 | Summarise key results with reference to study objectives *Discussion Paragraph 2 and Paragraph 3* |
| Limitations | 19 | Discuss limitations of the study, taking into account sources of potential bias or imprecision. Discuss both direction and magnitude of any potential bias: *Abstract and* *Discussion Paragraph 4* |
| Interpretation | 20 | Give a cautious overall interpretation of results considering objectives, limitations, multiplicity of analyses, results from similar studies, and other relevant evidence *Discussion Paragraph 2, Paragraph 3, Paragraph 4, Pargraph 5.* |
| Generalisability | 21 | Discuss the generalisability (external validity) of the study results *Discussion, Paragraph 3 and Paragraph 5* |
| Other information | | |
| Funding | 22 | Give the source of funding and the role of the funders for the present study and, if applicable, for the original study on which the present article is based *See financial disclosure* |

*Give information separately for cases and controls in case-control studies and, if applicable, for exposed and unexposed groups in cohort and cross-sectional studies.

**Note:** An Explanation and Elaboration article discusses each checklist item and gives methodological background and published examples of transparent reporting. The STROBE checklist is best used in conjunction with this article (freely available on the Web sites of PLoS Medicine at http://www.plosmedicine.org/, Annals of Internal Medicine at http://www.annals.org/, and Epidemiology at http://www.epidem.com/). Information on the STROBE Initiative is available at www.strobe-statement.org.
