# Supplementary material for: Hepatitis B and hepatitis D virus infections in the Central African Republic, twenty-five years after a fulminant hepatitis outbreak, indicate continuing spread in asymptomatic young adults
Source: PLoS Negl Trop Dis. 2018 Apr 26;12(4):e0006377. doi: 10.1371/journal.pntd.0006377 (PMC5940242; doi:10.1371/journal.pntd.0006377)
Supplement: S1 Table — (DOC) [file pntd.0006377.s002.doc]

**S1 Table 1: Age, marital status, district address in Bangui, and blood borne and sexual risk factors in students and pregnant women in Central Africa Republic (CAR) in year 2010**

|  |  | **Students (n=1298)** | **Pregnant women (n=874)** | ***P*** |
| --- | --- | --- | --- | --- |
| Age (years; mean) [SD] |  | 21.87 ± 4.03 | 25.01 ± 5.74 | <0.001 |
| Female:male |  | 448:848 | 874:0 | <0.001 |
| Marital status (n/%) |  |  |  | <0.001 |
| (n=1296;874) | Single | 1195 (92.2%) | 644 (73.6%) |  |
|  | Live-in partnership | 81 (6.3%) | 102 (11.7%) |  |
|  | Married monogamous  Married polygamous | 18 (1.4%)  0,0 (0.0%) | 90 (10.3%)  37(4.2%) |  |
|  | Widowed | 2 (0.2%) | 2 (0.2%) |  |
| District Address in Bangui |  | See details in S3 Table 3 | | <0.001 |
| CAR nationality |  | 1281 (98.6%) | 843 (96.4%) | <0.001 |
| Risk factors | Previous viral hepatitis  (n=1041;859) | 35 (3.0%) | 8 (0.9%) | <0.002 |
|  | Previous Icterus  (n=1286;871) | 154 (12.0%) | 33 (3.8%) | <0.001 |
| (n=1298;874) | Surgery | 96 (7.4%) | 74 (8.5%) | 0.372 |
|  | Dental extraction | 274 (21.1%) | 359 (41.0%) | <0.001 |
|  | Blood transfusion | 62 (4.8%) | 39 (4.5%) | 0.802 |
|  | Tatoo | 56 (4.3%) | 82 (9.4%) | <0.001 |
|  | Intravenous drug user | 10 (0.8%) | 1 (0.1%) | 0.029 |
|  | Sharp-edged tool use | 771 (59.5%) | 355 (40.6%) | <0.001 |
|  | Alcohol  Multiple partners before  Multiple partners in 2010  Use of condom always  sometimes  never | 618 (47.7%)  471 (36.3%)  124 (9.6%)  558  515  136 | 435 (49.7%)  311 (35.5%)  9 (1.0%)  3  0  872 | 0.353  0.703  <0.001  <0.001 |
| Previous HBV vaccination | (n=1293:872) | 20 (1.5%) | 23 (2.6%) | 0.074 |
