# Supplementary material for: Hepatitis B and hepatitis D virus infections in the Central African Republic, twenty-five years after a fulminant hepatitis outbreak, indicate continuing spread in asymptomatic young adults
Source: PLoS Negl Trop Dis. 2018 Apr 26;12(4):e0006377. doi: 10.1371/journal.pntd.0006377 (PMC5940242; doi:10.1371/journal.pntd.0006377)
Supplement: S2 Table — This list corresponds to almost all different high educational entities in Bangui, Central African Republic. (DOC) [file pntd.0006377.s003.doc]

**S2 Table 2: Students repartition among high schools and University departments. This list corresponds to almost all different high educational entities in Bangui, Central African Republic.**

| Establishment | Volunteers number for the study | Percentage |
| --- | --- | --- |
| Centre Préparatoire Internationale | 14 | 1.1 |
| Centre Protestant pour la Jeunesse | 41 | 3.2 |
| Ecole Normale Supérieure | 36 | 2.8 |
| Faculté Economie et Droit | 138 | 10.7 |
| Faculté des Lettres et Sciences Humaines | 65 | 5.0 |
| Faculté des Sciences de la Santé | 36 | 2.8 |
| Faculté des Sciences | 54 | 4.2 |
| Institut Universitaire de Technologie | 22 | 1.7 |
| Facs autres | 29 | 2.2 |
| Institut de gestion | 8 | 0.6 |
| Lycée Bimbo | 52 | 4.0 |
| Lycée Boganda | 147 | 11.4 |
| Lycée Caron | 76 | 5.9 |
| Lycée Fatima | 43 | 3.3 |
| Lycée Gobongo | 63 | 4.9 |
| Lycée Jean Marie | 8 | 0.6 |
| Lycée des martyrs | 197 | 15.3 |
| Lycée Pie XII | 22 | 1.7 |
| Lycée Rapide | 35 | 2.7 |
| Lycée Saint Charles | 7 | 0.5 |
| Lycée technique | 79 | 6.1 |
| Lycée Miskine | 93 | 7.2 |
| Lycée New Tech | 26 | 2.0 |
| Total | 1291 | 100.0 |
