# Supplementary material for: Hepatitis B and hepatitis D virus infections in the Central African Republic, twenty-five years after a fulminant hepatitis outbreak, indicate continuing spread in asymptomatic young adults
Source: PLoS Negl Trop Dis. 2018 Apr 26;12(4):e0006377. doi: 10.1371/journal.pntd.0006377 (PMC5940242; doi:10.1371/journal.pntd.0006377)
Supplement: S3 Table — The gray scale indicates the economical status of the district. (DOC) [file pntd.0006377.s004.doc]

**S3 Table 3:** **Place of living of the studied cohort in the different districts (1-8) of Bangui capital and Bimbo suburbs district in function of HBV and HDV status. The gray scale indicates the economical status of the district.**

| District in Bangui capital | HBsAg and/or Anti-HBc Abs (n = 2162) | | *p* | | HDAg and/or Anti HD Abs  (n = 181) | | | *p* | | Cohorts  (n=2170) | | | *p* | |  |
| --- | --- | --- | --- | --- | --- | --- | --- | --- | --- | --- | --- | --- | --- | --- | --- |
|  | negative | positive | | 0.769 | | negative | positive | | 0.165 | | Students | Pregnant Women | | <0.001 | |
| 1 | 28 (70.0%) | 12 (30.0%) | |  | | 3 (75.0%) | 1 (25.0%) | |  | | 38 (2.9%) | 2 (0.2%) | |  | |
| 2 | 151 (80.3%) | 37 (19.7%) | |  | | 9 (81.8%) | 2 (18.2%) | |  | | 137 (10.6%) | 51 (5.8%) | |  | |
| 3 | 167 (73.2%) | 59 (25.9%) | |  | | 21 (95.5%) | 1 (4.5%) | |  | | 117 (9.0%) | 111 (12.7%) | |  | |
| 4 | 324 (74.7%) | 109 (25.1%) | |  | | 40 (97.6) | 1 (2.4%) | |  | | 234 (18.1%) | 200 (22.9%) | |  | |
| 5 | 429 (75.5%) | 135 (23.8%) | |  | | 36 (80.0% | 9 (20.0%) | |  | | 285 (22.0%) | 282 (32.3%) | |  | |
| 6 | 146 (71.9%) | 56 (27.6%) | |  | | 16 (88.9%) | 2 (11.1%) | |  | | 169 (13.0%) | 34 (3.9%) | |  | |
| 7 | 34 (77.3%) | 10 (22.7%) | |  | | 3 (100.0%) | 0 (0.0%) | |  | | 40 (3.1%) | 4 (0.5%) | |  | |
| 8 | 195 (74.7%) | 65 (24.9%) | |  | | 19 (90.5%) | 2 (9.5%) | |  | | 140 (10.8%) | 121 (13.8%) | |  | |
| Bimbo | 157 (76.6%) | 48 (23.4%) | |  | | 16 (94.1%) | 1 (5.9%) | |  | | 136 (10.5%) | 69 (7.9%) | |  | |
| Total | 1631 (75.4%) | 531 (24.6%) | |  | | 163 (89.6%) | 19 (10.4%) | |  | | 1296 | 874 | |  | |

Low socio-economical district High socio-economical district
