# Supplementary material for: Hepatitis B and hepatitis D virus infections in the Central African Republic, twenty-five years after a fulminant hepatitis outbreak, indicate continuing spread in asymptomatic young adults
Source: PLoS Negl Trop Dis. 2018 Apr 26;12(4):e0006377. doi: 10.1371/journal.pntd.0006377 (PMC5940242; doi:10.1371/journal.pntd.0006377)
Supplement: S4 Table — (DOC) [file pntd.0006377.s005.doc]

**S4 Table 4: Occupation of subjects from the 2 cohorts (student and pregnant women) in function of HBV and HDV status.**

| Occupation | HBsAg and/or Anti-HBc Abs  (n = 2132) | | *p* | HDAg and/or Anti HD Abs  (n = 181) | | *p* |
| --- | --- | --- | --- | --- | --- | --- |
|  | negative | positive | 0.593 | negative | positive | 0.119 |
| Student | 1208 (74.7%) | 410 (25.3%) |  | 127 (92.7%) | 10 (7.3%) |  |
| Housewife | 263 (78.5%) | 72 (21.5%) |  | 21 (80.8%) | 4 (16.7%) |  |
| Teacher | 11 (68.8%) | 5 (31.3%) |  | 1 (100.0%) | 0 (0.0%) |  |
| Health care workers | 10 (76.9%) | 3 (23.1%) |  | 2 (66.7%) | 1 (33.3%) |  |
| Other | 114 (76.0%) | 36 (24.0%) |  | 12 (80.0%) | 3 (20.0%) |  |
| Total | 1606 (75.4%) | 526 (24.6) |  | 163 (90.0%) | 18 (10.0%) |  |
