# Supplementary material for: Hepatitis B and hepatitis D virus infections in the Central African Republic, twenty-five years after a fulminant hepatitis outbreak, indicate continuing spread in asymptomatic young adults
Source: PLoS Negl Trop Dis. 2018 Apr 26;12(4):e0006377. doi: 10.1371/journal.pntd.0006377 (PMC5940242; doi:10.1371/journal.pntd.0006377)
Supplement: S5 Table — (DOCX) [file pntd.0006377.s006.docx]

**S5 Table 5: Comparison of HBV status among 1296 young asymptomatic students in Bangui, Central Africa Republic (CAR) in 2010**

| **Anti-HBc antibodies AND/OR HBsAg** | | **Negative (n=947)** | **Positive (n=349)** | ***p*** |
| --- | --- | --- | --- | --- |
| Age (years; mean ± SD) |  | 21.7 ± 3.9 | 22.3 ± 4.5 | 0.049 |
|  |  |  |  |  |
| Female:Male |  | 313:634 | 135:214 | 0.059 |
|  |  |  |  |  |
| Marital status (n/%) |  |  |  | 0.787 |
|  | Single | 872 (92.1%) | 323 (92.6%) |  |
|  | Live-in partnership | 61 (6.4%) | 20 (5.7%) |  |
|  | Married (monogamous) | 13 (1.4%) | 5 (1.4%) |  |
|  | Married (polygamous) | 0 (0%) | 0 (0%) |  |
|  | Widowed | 1 (0.1%) | 1 (0.3%) |  |
|  |  |  |  |  |
| CAR nationality |  | 937 (98.9%) | 344 (98.6%) | 0.574 |
|  |  |  |  |  |
| Risk factors | Previous viral hepatitis (n = 1186) | 24 (2.8%) | 11 (3.4%) | 0.580 |
|  | Previous icterus (n = 1286) | 105 (11.2%) | 49 (14.1%) | 0.157 |
|  | Surgery (n = 1296) | 68 (7.2%) | 28 (8.0%) | 0.608 |
|  | Dental extraction (n = 1293) | 204 (21.6%) | 70 (20.1%) | 0.619 |
|  | Blood transfusion (n = 1292) | 49 (5.2%) | 13 (3.7%) | 0.348 |
|  | Tattoo (n = 1296) | 36 (3.8%) | 20 (5.7%) | 0.130 |
|  | Intravenous drug use (n = 1296) | 7 (0.7%) | 3 (0.9%) | 0.734 |
|  | Sharp-edged tool use (n = 1296) | 567 (59.9%) | 204 (58.5%) | 0.644 |
|  | Alcohol (n = 1296) | 458 (48.4%) | 160 (45.8%) | 0.421 |
|  | Multiple partners previously (n = 1296) | 339 (35.8%) | 132 (37.8%) | 0.501 |
|  | Multiple partners in 2010 (n = 1296) | 93 (9.8%) | 31 (8.9%) | 0.611 |
|  | Use of condoms (n = 1209) |  |  | 0.822 |
|  | -always | 412 (46.6%) | 146 (44.9%) |  |
|  | -sometimes | 375 (42.4%) | 140 (43.1%) |  |
|  | -never | 97 (11.0%) | 39 (12.0%) |  |
|  |  |  |  |  |
| Previous HBV vaccination | (n = 1293) | 15 (1.6%) | 5 (1.4%) | 0.846 |
