# Supplementary material for: Hepatitis B and hepatitis D virus infections in the Central African Republic, twenty-five years after a fulminant hepatitis outbreak, indicate continuing spread in asymptomatic young adults
Source: PLoS Negl Trop Dis. 2018 Apr 26;12(4):e0006377. doi: 10.1371/journal.pntd.0006377 (PMC5940242; doi:10.1371/journal.pntd.0006377)
Supplement: S6 Table — (DOCX) [file pntd.0006377.s007.docx]

**S6 - Table 6: Comparison of HBV status among 866 young asymptomatic pregnant women in Bangui, Central Africa Republic (CAR) in 2010**

| **Anti-HBc antibodies AND/OR HBsAg** | | **Negative (n=684)** | **Positive (n=182)** | ***p*** |
| --- | --- | --- | --- | --- |
| Age (years; mean ± SD) | | 25.0 ± 5.8 | 25.2 ± 5.5 | 0.587 |
|  |  |  |  |  |
| Marital status (n/%) |  |  |  | 0.084 |
|  | Single | 506 (74.0%) | 131 (72.0%) |  |
|  | Live-in partnership | 72 (10.5%) | 30 (16.5%) |  |
|  | Married (monogamous) | 76 (11.1%) | 13 (7.1%) |  |
|  | Married (polygamous) | 29 (4.2%) | 7 (3.8%) |  |
|  | Widowed | 1 (0.1%) | 1 (0.5%) |  |
|  |  |  |  |  |
| CAR nationality |  | 657 (96.1%) | 177 (97.3%) | 0.446 |
|  |  |  |  |  |
| Risk factors | Previous viral hepatitis (n = 859) | 3 (0.4%) | 4 (2.2%) | 0.039 |
|  | Previous icterus (n = 862) | 27 (4.0%) | 6 (3.3%) | 0.685 |
|  | Surgery (n = 866) | 58 (8.5%) | 16 (8.8%) | 0.894 |
|  | Dental extraction (n = 862) | 287 (42.1%) | 68 (37.6%) | 0.305 |
|  | Blood transfusion (n = 860) | 26 (3.8%) | 13 (7.2%) | 0.084 |
|  | Tattoo (n = 866) | 66 (9.6%) | 15 (8.2%) | 0.562 |
|  | Intravenous drug use (n = 866) | 1 (0.1%) | 0 (0.0%) | 1.000 |
|  | Sharp-edged tool use (n = 866) | 284 (41.5%) | 66 (36.3%) | 0.199 |
|  | Alcohol (n = 866) | 326 (47.7%) | 104 (57.1%) | 0.023 |
|  | Multiple partners previously (n = 866) | 240 (35.1%) | 69 (37.9%) | 0.480 |
|  | Multiple partners in 2010 (n = 866) | 5 (0.7%) | 4 (2.2%) | 0.099 |
|  | Use of condoms (n = 866) |  |  | 0.508 |
|  | - always | 2 (0.3%) | 1 (0.5%) |  |
|  | - sometimes | 0 (0%) | 0 (0%) |  |
|  | -never | 682 (99.7%) | 181 (99.5%) |  |
|  |  |  |  |  |
| Previous HBV vaccination | (n = 863) | 16 (2.3%) | 6 (3.3%) | 0.433 |
