# Supplementary material for: Hepatitis B and hepatitis D virus infections in the Central African Republic, twenty-five years after a fulminant hepatitis outbreak, indicate continuing spread in asymptomatic young adults
Source: PLoS Negl Trop Dis. 2018 Apr 26;12(4):e0006377. doi: 10.1371/journal.pntd.0006377 (PMC5940242; doi:10.1371/journal.pntd.0006377)
Supplement: S7 Table — (DOCX) [file pntd.0006377.s008.docx]

**S7 Table 7: Comparison of HDV status among 113 HBsAg-positive students in Bangui, Central Africa Republic (CAR) in 2010**

| **HDV (Anti-HD AND/OR HDAg)** | | **Negative (n=107)** | **Positive (n=6)** | ***p*** |
| --- | --- | --- | --- | --- |
| Age (years;mean ± SD) |  | 22.3 ± 4.6 | 25.2 ± 9.0 | 0.167 |
|  |  |  |  |  |
| Female:Male |  | 47:60 | 4:2 | 0.407 |
|  |  |  |  |  |
| Marital status (n/%) |  |  |  | 0.498 |
|  | Single | 96 (89.7%) | 5 (83.3%) |  |
|  | Live-in partnership | 8 (7.5%) | 1 (16.7%) |  |
|  | Married (monogamous) | 2 (1.9%) | 0 (0.0%) |  |
|  | Married (polygamous) | 0 (0.0%) | 0 (0.0%) |  |
|  | Widowed | 1 (0.9%) | 0 (0.0%) |  |
|  |  |  |  |  |
| CAR nationality |  | 104 (97.2%) | 6 (100.0%) | 1.000 |
|  |  |  |  |  |
| Risk factors | Previous viral hepatitis (n=105) | 3 (3.0%) | 0 (0.0%) | 1.000 |
|  | Previous icterus (n=113) | 13 (12.1%) | 1 (16.7%) | 0.557 |
|  | Surgery (n= 113) | 4 (3.7%) | 0 (0.0%) | 1.000 |
|  | Dental extraction (n=113) | 18 (16.8%) | 1 (16.7%) | 1.000 |
|  | Blood transfusion (n=113) | 2 (1.9%) | 1 (16.7%) | 0.152 |
|  | Tattoo (n=113) | 7 (6.5%) | 2 (33.3%) | 0.072 |
|  | Intravenous drug use (n=113) | 1 (0.9%) | 0 (0.0%) | 1.000 |
|  | Sharp-edged tool use (n=113) | 63 (58.9%) | 3 (50.0%) | 0.692 |
|  | Alcohol (n=113) | 49 (45.8%) | 3 (50.0%) | 1.000 |
|  | Multiple partners previously (n=113) | 46 (43.0%) | 1 (16.7%) | 0.398 |
|  | Multiple partners in 2010 (n=113) | 11 (10.3%) | 0 (0.0%) | 1.000 |
|  | Use of condoms (n=109) |  |  | 0.216 |
|  | - always | 43 (41.7%) | 5 (83.3%) |  |
|  | - sometimes | 47 (45.6%) | 1 (16.7%) |  |
|  | - never | 13 (12.6%) | 0 (0.0%) |  |
| Previous HBV vaccination | (n=112) | 0 (0.0%) | 0 (0.0%) | - |
