# Supplementary material for: Hepatitis B and hepatitis D virus infections in the Central African Republic, twenty-five years after a fulminant hepatitis outbreak, indicate continuing spread in asymptomatic young adults
Source: PLoS Negl Trop Dis. 2018 Apr 26;12(4):e0006377. doi: 10.1371/journal.pntd.0006377 (PMC5940242; doi:10.1371/journal.pntd.0006377)
Supplement: S8 Table — (DOCX) [file pntd.0006377.s009.docx]

**S8 Table 8: Comparison of HDV status among 69 HBsAg-positive pregnant women in Bangui, Central Africa Republic (CAR) in 2010**

| **HDV (Anti-HD AND/OR HDAg)** | | **Negative (n=56)** | **Positive (n=13)** | ***p*** |
| --- | --- | --- | --- | --- |
| Age (years; mean + SD) |  | 25.1 ± 5.6 | 26.2 ± 4.0 | 0.508 |
|  |  |  |  |  |
| Marital status (n/%) |  |  |  | 1.000 |
|  | Single | 43 (76.8%) | 11 (84.6%) |  |
|  | Live-in partnership | 7 (12.5%) | 1 (7.7%) |  |
|  | Married (monogamous) | 3 (5.4%) | 1 (7.7%) |  |
|  | Married (polygamous) | 2 (3.6%) | 0 (0.0%) |  |
|  | Widowed | 1 (1.8%) | 0 (0.0%) |  |
|  |  |  |  |  |
| CAR nationality |  | 56 (100.0%) | 12 (92.3%) | 0.188 |
|  |  |  |  |  |
| Risk factors | Previous viral hepatitis (n=69) | 2 (3.6%) | 2 (15.4%) | 0.158 |
|  | Previous icterus (n=69) | 2 (3.6%) | 1 (7.7%) | 0.471 |
|  | Surgery (n= 69) | 8 (14.3%) | 1 (7.7%) | 1.000 |
|  | Dental extraction (n=69) | 22 (39.3%) | 7 (53.8%) | 0.338 |
|  | Blood transfusion (n=69) | 3 (5.4%) | 2 (15.4%) | 0.235 |
|  | Tattoo (n=69) | 4 (7.1%) | 2 (15.4%) | 0.315 |
|  | Intravenous drug use (n=69) | 0 (0.0%) | 0 (0.0%) | - |
|  | Sharp-edged tool use (n=69) | 22 (39.3%) | 5 (38.5%) | 1.000 |
|  | Alcohol (n=69) | 32 (57.1%) | 7 (53.8%) | 1.000 |
|  | Multiple partners previously (n=69) | 21 (37.5%) | 5 (38.5%) | 1.000 |
|  | Multiple partners in 2010 (n=69) | 2 (3.6%) | 1 (7.7%) | 0.471 |
|  | Use of condoms (n=69) |  |  | - |
|  | - always | 0 (0.0%) | 0 (0.0%) |  |
|  | - sometimes | 0 (0.0%) | 0 (0.0%) |  |
|  | - never | 56 (100%) | 13 (100.0%) |  |
| Previous HBV vaccination | (n=69) | 0 (0.0%) | 1 (7.7%) | 0.188 |
