# Supplementary material for: Hepatitis B and hepatitis D virus infections in the Central African Republic, twenty-five years after a fulminant hepatitis outbreak, indicate continuing spread in asymptomatic young adults
Source: PLoS Negl Trop Dis. 2018 Apr 26;12(4):e0006377. doi: 10.1371/journal.pntd.0006377 (PMC5940242; doi:10.1371/journal.pntd.0006377)
Supplement: S9 Table — HDV sequences from this study Accession Summary ----------------- Type Accession Unique Name Study PRJEB24597 ena-STUDY-Research Cancer Center of Lyon-24-01-2018-10:05:53:847–48 European Nucleotide Archive (ENA). (DOC) [file pntd.0006377.s010.doc]

**S9 Table 9: HDV sequences from this study, with accession number of the study**

Accession Summary -----------------

Type Accession

Unique Name Study PRJEB24597

ena-STUDY-Research Cancer Center of Lyon-24-01-2018-10:05:53:847-48

European Nucleotide Archive (ENA)

*HDV FH-CAR1985 clones:*

FH4-CAR1985cl1,2,3,4

FH7-CAR1985cl3,8,9,10

FH12-CAR1985cl2,3,4,5,6

FH27-CAR1985cl1,2,3,4

FH39-CAR1985cl1,2,3,6,7,9;

FH74-CAR1985cl4

FH82-CAR1985cl1S,2,3,4,4S

FH88-CAR1985cl1,2,3,4

FH111-CAR1985cl1,2,2S,3,4,4S

FH122-CAR1985cl1,4

FH123-CAR1985cl2

FH124-CAR1985cl1,3,4

*Student CAR2010 HDV direct sequences*

s148; d148

s180; d180

s288; d288

s349; d349

s525; d525

s569

s582; d582
