# Supplementary material for: Hepatitis B and hepatitis D virus infections in the Central African Republic, twenty-five years after a fulminant hepatitis outbreak, indicate continuing spread in asymptomatic young adults
Source: PLoS Negl Trop Dis. 2018 Apr 26;12(4):e0006377. doi: 10.1371/journal.pntd.0006377 (PMC5940242; doi:10.1371/journal.pntd.0006377)
Supplement: S1 Method — (DOCX) [file pntd.0006377.s013.docx]

**S1 Method:**

**Questionnaire**

**Participant inclusion Nº: |___|___|___|___|**

**SURVEY ON THE PREVALENCE OF VIRAL HEPATITIS DELTA IN CENTRAL AFRICAN REPUBLIC**

🙞 🙞 🙞 🙞 🙞

**Consent to participation**

I freely accept to give my consent to the terms of the study, performed by the Institut Pasteur de Bangui and the Centre Hospitalier & Universitaire de Bobigny (France), on the viral hepatitis B - Delta in Central African Republic. Recognizing that even after signing this document, I can at any time decide not to continue to participate in this study, that will not affect the rendering quality of the results or the benefits that I can get.

**1 – Full Name of the participant :____________________________________**

**Phone number of the participant** |___|___|___|___|___|___|___|___|___|

**Date : Day |___|___| Month |___|___| Year |___|___|___|___|**

**Signature :**

**2 – Parental permission (for minors and infants) :**

**Parent Full Name _________________________________________________**

**Date |__|__| |__|__| |__|__|__|__|**

**Signature Parental consent:**

**Parent Phone Number:** |___|___|___|___|___|___|___|___|___|

**3 – Name of Investigator who directed the blood collection: ______________________________**

**Date : Day |___|___| Month |___|___| Year |___|___|___|___|**

**Signature :**

**Relative Full Name and Phone number: _____________________________**

|___|___|___|___|___|___|___|___|___|

**SHEET OF INQUIRY**

**Codification**

**IDENTIFICATION**

**Date of birth** : /**______/______/__________/**

**Gender** : Male (1), female (2) **|___|**

**Occupation:** …………………………………………………………………………………………

**School/University** : ………………………………………………………………………………

**Class**: ………………………………………………………………………………………………

**District**: …………………………………………………………………………………………….

**Marital statut:** Married: Monogamy (1), Polygamy (2) ;

Singles (3), Widow (4), Cohabitation (5) |__|

**Nationality** : Central African Citizen (1), Foreign (2) |__|

**Specify if foreigner:** ………………………………………………

**BACKGROUNDS**

***1. Personnals***

**Viral hepatitis:** Yes (1), No (2) **|___|**

**Jaundice**: Yes (1), No (2) **|___|**

**Vaccine against hepatitis B** : Yes (1), No (2) **|___|**

**Surgery**: Yes (1), No (2) **|___|**

**Tooth extraction**: Yes (1), No (2) **|___|**

**Blood transfusion**: Yes (1), No (2) **|___|**

**2. *Others specify***: …………………………………………………………………………………………………………………………………………………………………………………………………………………………

…………………………………………………………………………………………………………..

**INFORMATION**

**1 – Sexual Life (Not applicable to newborns and infants 0 – 6 months) :**

**Do you have multiple sex partners in the past**: Yes (1), No (2) **|___|**

If yes, can you give an approximate number? ……………….

**Do you have now multiple sex partners**: Yes (1), No (2) **|___|**

If yes, can you give an approximate number?: ……………….

**During sex, do you use condoms**?

Always (1), Sometimes (2), Never (3) **|___|**

**2 – Others**

**Do you have any tattoos**: Yes (1), No (2) **|___|**

**Have you ever used injection drugs**: Yes (1), No (2) **|___|**

**Have you or do you share hardware:**

**cuting (scissors, blades, razor, etc.)**: Yes (1), No (2) **|___|**

**Do you consume alcoholic beverages**: Yes (1), No (2) **|___|**

**Others to be specified :** ……………………………………………………………………

**3 – Pregnant women**

**How many full-term pregnancies**:  **|___|**

**How many living children**:  **|___|**

**All children have the same parentage?** : Yes (1), No (2) **|___|**

**Interviewer Full Name: __________________________________**

**Serology**

**(*To be completed at the Institut Pasteur de Bangui*)**

1. **HBsAg: 1. Positive |___| 2. Negative |___| 3. Confirmation |___|**
2. **Total anti-HBc antibodies: Positive |___| Negative |___|**
3. **Anti-HBc antibodies (IgM) : Positive |___| Negative |___|**
4. **HBeAg: Positive |___| Negative |___|**
5. **Anti-HBe antibodies: Positive |___| Negative |___|**
6. **Anti-HDV antibodies: Positive |___| Negative |___|**
7. **AgDelta : Positive |___| Negative |___|**

#### If HBsAg negative

1. **Anti-HBs antibodies: 1. < à 100 UI |___| 2. > à 100 UI |___|**

**4 – Newborns from HBsAg positive women (*To be completed by the Pediatrician*)**

- **APGAR at the birth …………………………………………….**
- **Head circumference …………………………………………………..**
- **High …………………………………………………………………..**
- **Weight ………………………………………………………………….**
- **Evolutions after birth:**
  - **Died ……………………………………………………….**
  - **Living …………………………………………………………**
